# Comparison of the Reliability of Four Different Movement Thresholds When Evaluating Vertical Jump Performance

**DOI:** 10.3390/sports10120193

**Published:** 2022-11-29

**Authors:** Madeleine Barefoot, Hugh Lamont, J. Chadwick Smith

**Affiliations:** Neuromechanics Laboratory, Department of Kinesiology, Coastal Carolina University, Conway, SC 29526, USA

**Keywords:** kinetics, kinematics, force platform, reliability

## Abstract

Analyzing vertical jumps performed on a force plate can be useful for the strength and conditioning professional in managing neuromuscular fatigue. The purpose of this study was to compare different movement thresholds when analyzing countermovement (CJ) and squat jump (SJ) performance. Twenty-one college-aged participants (9 female, 12 male) performed five CJs and five SJs. Movement initiation was identified when the vertical ground reaction force (VGRF) deviated five standard deviations (5SD), four standard deviations, (4SD), 2.5% of system weight (2.5%SW), and 10% of system weight (10%SW) from their starting position. For CJs, movement was determined when the VGRF deviated either above or below these thresholds (5SDAB, 4SDAB, 2.5%SWAB, 10%SWAB) and was compared to when VGRF deviated below these thresholds (5SDB, 4SDB, 2.5%SWB, 10%SWB) in terms of peak force (Fmax), net impulse (netIMP), braking impulse (brIMP), propulsive impulse, jump height (JHT), peak power (Pmax), peak velocity (Vmax), and RSImod. For SJs, movement was determined when VGRF initially rose above these thresholds (5SD, 4SD, 2.5%SW, and 10%SW) for Fmax, netIMP, JHT, and Vmax. Significant differences were observed among several methods except for Fmax. However, these differences were small. All CJ measures demonstrated good-to-excellent relative reliability (ICC: 0.790–0.990) except for netIMP for 2.5%SWAB (ICC: 0.479). All methods demonstrated good absolute reliability as measured by percent coefficient of variation (CV%) except brIMP and RSImod. This may be due to instructions given to each jumper as well as skill level. For SJs, no differences in Fmax or netIMP were found across all methods. Small differences were seen for JHT, Pmax, and Vmax across several methods. All methods produced acceptable CV% (<10%) and excellent ICCs (0.900–0.990). However, some jumpers produced CV% that was greater than 10% when determining JHT for 5SD, 4SD, and 2.5%SW methods. This could be due to our method of obtaining system weight. Based on our findings, we recommend using the 10%SW method for assessing SJ performance on a force plate.

## 1. Introduction

The countermovement jump (CJ) and squat jump (SJ) are two commonly employed tests used to monitor and assess athletic performance. Athletic monitoring and testing are essential to assist the strength and conditioning professionals with managing neuromuscular fatigue that is a consequence of the athlete’s training regimen. Vertical jump height can easily be obtained as a rudimentary measure of performance. When these tests are performed on a force plate, additional kinetic and kinematic variables can be obtained to identify important biomechanical factors related to performance [1,2,3,4,5,6].

A methodological concern with calculating many of these variables is being able to reliably determine the beginning of the jump movement. Several different methods have been employed to identify movement initiation in CJs. Previous methods used include manually identifying when the vertical ground reaction force (VGRF) deviates from baseline (i.e., VGRF during quiet stance) [7,8], identifying when the VGRF deviates a predetermined percentage of bodyweight from baseline [9,10,11,12], identifying when the VGRF deviates a predetermined number of standard deviations from baseline [13,14,15,16], and identifying when the VGRF drops below baseline by a predetermined magnitude of force (e.g., 10 N) [17].

The manual selection of the beginning of the movement may introduce more variability in determining several kinetic and kinematic variables. Thus, the validity of this method has been called into question [11,18]. Meylan et al. [10] evaluated different percentages of the subject’s body weight (2.5%, 5%, and 10%) to determine the beginning of CJs in calculating several kinematic and kinetic variables in ten soccer players. While all three methods demonstrated similar variability, using the 10% body weight criteria resulted in significant differences in their measures compared to the other two methods. The authors recommended using the 2.5% body weight criteria since it retained more of the force-time curve. This recommendation was justified by the unfiltered noise in the signal being slightly less than (14 N) 2.5% of body weight (~18 N). Owen et al. [14] evaluated the selection of sampling frequency, numerical integration method, and identification of the initiation of the CJ when assessing peak lower body power in male rugby players. They recommended using 1000 Hz as the sampling rate with either the Simpson’s rule or trapezoidal rule for integration, and identifying the beginning of the movement using body weight ± 5 SD but start integration of the force-time curve at 30 ms prior to the jumper exceeding the 5 SD threshold. Assessing other kinetic and/or kinematic variables may require a different threshold criteria. Perez-Castilla et al. [11] compared absolute methods (i.e., 10 N and 50 N) and relative methods (i.e., 1% system weight, 10% system weight, and 5 SD minus 30 ms) for determining several kinetic and kinematic variables produced during loaded CJs in male sports science students. They concluded that the 50 N, 10% system weight, and 5 SD minus 30 ms produced better reliability and were significantly different compared to the 10 N and 1% system weight thresholds, especially for variables measured during the eccentric phase. The mean body mass for their participants was 76.3 ± 7.5 kg. Presumably, using absolute methods (e.g., 10 N, 50 N) to identify movement in populations with less or more body mass could lead to errors in identifying movement initiation in unloaded CJs. The percentage of body weight methods have not been compared to the standard deviation methods for identifying movement initiation to measure a variety of kinetic and kinematic variables produced during unloaded CJs. Furthermore, there has been no comparison of movement initiation methods for SJs. Therefore, the purpose of this investigation was to compare different movement initiation methods for several kinetic and kinematic variables produced during both CJs and SJs.

## 2. Materials and Methods

### 2.1. Participants

Twenty-one participants (mean ± SD, female, *n* = 9, age, 21.4 ± 1.2 yrs; height, 165.4 ± 4.8 cm; body mass, 68.3 ± 19.5 kg; male, *n* = 12, age, 22.5 ± 3.3 years; height, 179.1 ± 5.5 cm, body mass, 84.7 ± 10.9 kg) voluntarily participated in this study. Four participants were considered sedentary (not participating in exercise more than once per week). Seventeen participants reported currently participating in resistance training and/or aerobic training at least twice per week. While this was a convenience sample, previous studies evaluating different movement thresholds used sample sizes ranging from 10 to 17 subjects [9,10,11,14]. All participants were free from any neurological or musculoskeletal condition or injury that would prohibit them from performing a maximal effort vertical jump. All procedures and protocols were approved by the University’s Institutional Review Board (Protocol #2022.99). All participants provided their written informed consent.

### 2.2. Procedures

Using a repeated measures design, all participants completed three sessions (familiarization, SJ, and CJ) for this study. All three sessions were scheduled at least 48 h apart and no more than one week apart with participants completing all three sessions at the same time of day. The familiarization session was completed first by all participants. The experimental sessions (SJ and CJ) were completed in a randomized order.

For the familiarization session, participants completed the informed consent and health history questionnaire followed by having their height and body mass measured. Following a five-minute warm-up on a motorized treadmill at a self-selected pace, participants performed several practice jumps while standing on a portable force plate (Kistler Type 9260AA6; Kistler Instruments AG, Winterthur, Switzerland). All jumps were performed with the participants’ hands on their hips. When practicing and performing the SJs, the force plate was positioned inside a squat rack. To standardize the starting position for each SJ, an elastic band was attached to the squat rack. Due to differences in physical activity levels, we anticipated differences in strength levels and jumping ability across our participants. Therefore, we did not have all participants obtain a 90° knee joint angle as their starting position in the SJs. Instead, we instructed the participants to obtain a comfortable deep squat position. The vertical distance of the elastic band from the force plate was measured to ensure the same starting position for all subsequent SJs during the familiarization session as well as the SJ session. For the CJs, the depth of the countermovement was not standardized. Participants were instructed to drop down to a depth that would help them achieve the maximum jump height. Participants were considered familiarized with the jumping procedure when they were able to perform each type of jump and land on the force plate without losing their balance and when the participant reported that they were comfortable with performing each jump.

For the SJ and CJ sessions, participants started each session with a five-minute warm-up at a self-selected pace on a motorized treadmill. The same speed was used for each session. At the conclusion of the five-minute warm-up, each participant was taken through a standardized dynamic warm-up (i.e., forward gate swings, high knees, and walking lunges) [19] over a distance of 14 m by one of the investigators. After the dynamic warm-up, each participant completed two sub-maximal (50% and 75%) effort vertical jumps on the force plate with 30 s of rest between each jump. Following a 30 s rest, participants completed five maximal effort vertical jumps with one-minute rest between each jump. For the SJ session, participants were instructed to squat down to the elastic band that was set at the height determined during the familiarization session. This position was held for approximately two seconds to remove the influence of the stretch-shortening cycle. Subjects were then given a countdown of “3, 2, 1, jump”. If we visually observed the participant moving downward before jumping upward or if the participant started moving prior to the “jump” command, the participant was instructed to do the attempt again after a one-minute rest.

### 2.3. Data Analysis

Force plate data were sampled at 1000 Hz and collected using BioWare (Version 5.4.8, Kistler Instruments AG). The time and VGRF data for each jump were exported as a text file to be analyzed offline (after data collection was completed) using a custom software program written in Python. For CJ files, peak force (Fmax), net impulse (netIMP), braking impulse (brIMP), propulsive impulse, jump height (JHT), peak power (Pmax), peak velocity (Vmax), and reactive strength index modified (RSImod) were analyzed based on each of the movement initiation methods. For the SJ files, Fmax, netIMP, JHT, Pmax, and Vmax were analyzed offline using each of the movement initiation methods.

System weight was determined by taking the mean VGRF over the first second of data collection during quiet stance on the force plate. The beginning of each jump (squat and countermovement) was identified using each of the following methods: VGRF deviated from the mean system weight by five standard deviations (5SD), VGRF deviated from the mean system weight by four standard deviations (4SD), VGRF deviated from the mean system weight by 10% (10%SW), and VGRF deviated from the mean system weight by 2.5% (2.5%SW). For CJs, each method was used to identify the initial deviation above or below these thresholds (5SDAB, 4SDAB, 10%SWAB, and 2.5%AB), whichever occurred first, and the initial deviation below each threshold (5SDB, 4SDB, 10%SWB, and 2.5%SWB). When calculating jump height using the impulse-momentum method, the initial velocity is assumed to be zero. During CJs, the VGRF may initially move above the threshold before moving below, indicating that the participant elevated their center of mass by standing on their toes. This movement would be ignored if the movement was identified beginning when the VGRF dropped below the requisite criteria. This would also indicate that the initial velocity was not zero. Therefore, some error may be introduced into the calculation of jump height using the impulse-momentum method since the initial velocity would not be zero. In the calculation of the kinetic and kinematic variables (see next section), if the VGRF initially rose above the requisite threshold, this part of the movement was included in the unweighing phase of the CJ. Each kinetic and kinematic variable was calculated for each of the five jumps for each session. For each method of analysis, each kinetic and kinematic variable was averaged across all five trials for each participant. These means were used for statistical analysis. Movement initiation was determined when the VGRF exceeded each of these thresholds for the SJ session.

### 2.4. Calculation of Kinetic and Kinematic Variables

The force-time curve was evaluated from the beginning of the movement based on the corresponding movement initiation method and ending at take-off, which was identified as the first occurrence when the VGRF dropped below 10 N. Fmax was determined by the maximum amount of force produced during the jump. Net force was determined by subtracting system weight (i.e., the subject’s body weight) from the VGRF. NetIMP was determined by integrating the net force and time from movement initiation to take-off. For the 5SD methods, integration of the net force and time began 30 ms prior to the 5SD threshold [14]. JHT was determined using the impulse-momentum method. Acceleration-time curve was calculated by dividing the net-force by the participant’s body mass. Velocity-time curve was calculated from the integral of acceleration and time. Vmax was determined by the maximum velocity produced during the jump. Position-time curve was calculated from the integral of velocity and time. Power-time curve was calculated by the product of VGRF and velocity. Pmax was determined by the maximum power produced during the jump.

For the CJs, three additional variables were calculated, brIMP, propulsive impulse, and RSImod. The force-time curve was separated into braking and propulsive phases to identify impulse produced during each phase. The beginning of the braking phase was identified when the velocity was the lowest and the end of the braking phase was identified when the velocity was zero [18]. BrIMP was determined by the product of the average net-force and duration of time for this phase. The propulsive phase was determined by the end of the braking phase and take-off [18]. The propulsive impulse was determined by the product of the average net-force and duration of time for this phase. RSImod was calculated by dividing jump height by time from movement initiation to take-off [20].

### 2.5. Statistical Analysis

Separate one-way repeated measures ANOVA were used to analyze differences in the previously mentioned kinetic and kinematic variables across the eight different movement initiation methods for the CJs. For the SJs, separate one-way repeated measures ANOVA were used to assess differences in the above mentioned kinetic and kinematic variables across the four different movement initiation methods. Coefficient of variation (CV%) was calculated for each participant for each type of movement initiation method, jump type, and kinetic and kinematic variables. CV% was determined for each participant using the equation below:

$$\mathrm{CV}\%=\frac{Standard\;Deviation}{Mean}\times 100$$

The threshold for acceptable CV% was set to 10% [9].Relative reliability was determined for each movement threshold for CJs and SJs using intraclass correlation coefficients (ICCs) using a two-way mixed effects model for single measures. ICCs and their 95% CI were interpreted using the following scale: <0.5, poor; between 0.5 and 0.75, moderate; between 0.75 and 0.9, good; >0.90, excellent reliability [21]. All statistical analyses were performed using SPSS (Version 28.0; IBM Corp., Armonk, NY, USA). If sphericity was violated, Greenhouse-Geisser correction was used in subsequent analyses. Pairwise comparisons with a Bonferroni adjustment were used as a post hoc test when the results of the repeated measures ANOVA detected significant differences across the different movement initiation methods. Effect sizes were calculated by hand using Hedges’ g. Magnitude of effect size was interpreted using the following scale: 0.0 to 0.2, trivial; 0.2 to 0.6, small; 0.6 to 1.2, moderate; 1.2 to 2.0, large; 2.0 to 4.0, very large; 4.0+, nearly perfect [22]. Statistical significance was set a priori to *p* < 0.05.

## 3. Results

The results from each kinetic and kinematic variable and CV% assessed for each movement initiation method during the CJs are listed in Table 1 and Table 2, respectively. Table 3 lists the ICCs for the CJ data. There were no differences in Fmax across the different movement thresholds. The repeated measures ANOVA for netIMP showed a significant difference across the movement thresholds for CJs (F = 4.459, *p* = 0.029). Pairwise comparisons revealed the 5SDB produced a smaller netIMP compared to 10%SWAB (*p* = 0.009, ES = 0.03) and 10%SWB (*p* = 0.036, ES = 0.02). The netIMP for 4SDB was also smaller compared to 10%SWAB (*p* = 0.012, ES = 0.03) and 10%SWB (*p* = 0.047, ES = 0.03). The netIMP for 2.5%SWB was also smaller compared to 10%SWB (*p* = 0.016, ES = 0.03).

The repeated measures ANOVA for brIMP revealed significant differences across the movement thresholds for brIMP (F = 9.474, *p* = 0.002) for CJs. The brIMP for 5SDB was larger compared to 10%SWAB (*p* = 0.001, ES = 0.11) and 10%SWB (*p* = 0.001, ES = 0.11). The 4SDB also had a larger brIMP compared to the 10%SWAB (*p* = 0.001, ES = 0.11) and 10%SWB (*p* = 0.001, ES = 0.10). The 2.5%SWB also had a larger brIMP compared to 10%SWAB (*p* = 0.001, ES = 0.10) and 10%SWB (*p* = 0.001, ES = 0.10).

The repeated measures ANOVA for propulsive impulse showed a significant difference across movement thresholds for CJs (F = 9.417, *p* = 0.002). The 5SDB propulsive impulse was significantly lower compared to 10%SWAB (*p* = 0.001, ES = 0.05) and 10%SWB (*p* = 0.001, ES = 0.05). Similarly, the 4SDB propulsive impulse was also lower than the 10%SWAB (*p* = 0.001, ES = 0.04) and 10%SWB (*p* = 0.001, ES = 0.04) counterparts. The 2.5%SWB propulsive impulse was also lower compared to the 10%SWAB (*p* = 0.001, ES = 0.04) and 10%SWB (*p* = 0.001, ES = 0.04) counterparts.

The repeated measures ANOVA for JHT showed significant differences existed across the movement thresholds (F = 9.002, *p* = 0.003). Pairwise comparisons revealed the 5SDB JHT was lower than the 10%SWAB (*p* < 0.001, ES = 0.09) and 10%SWB JHT (*p* < 0.001, ES = 0.08). JHT for 4SDB was also lower than the JHT for 10%SWAB (*p* < 0.001, ES = 0.08) and 10%SWB (*p* < 0.001, ES = 0.08). The JHT for 2.5%SWB was also lower than the JHT for 10%SWAB (*p* < 0.001, ES = 0.08) and 10%SWB (*p* < 0.001, ES = 0.08).

The repeated measures ANOVA for Pmax showed significant differences existed across the movement thresholds (F = 10.641, *p* = 0.001) for CJs. Post hoc tests revealed Pmax for 10%SWAB was significantly greater than Pmax for 5SDB (*p* < 0.001, ES = 0.005), 4SDB (*p* < 0.001, ES = 0.004), and 2.5%SWB (*p* < 0.001, ES = 0.004). Pmax for 10%SWB was also greater than Pmax for 5SDB (*p* < 0.001, ES = 0.004), 4SDB (*p* < 0.001, ES = 0.004), and 2.5%SWB (*p* < 0.001, ES = 0.004).

The repeated measures ANOVA for Vmax showed significant differences existed across the movement thresholds (F = 11.358, *p* = 0.001) for CJs. Post hoc tests revealed Vmax was greater for 10%SWAB compared to Vmax for 5SDB (*p* < 0.001, ES = 0.009), 4SDB (*p* < 0.001, ES = 0.09), and 2.5%SWB (*p* < 0.001, ES = 0.08). Vmax for 10%SWB was also greater compared to Vmax for 5SDB (*p* < 0.001, ES = 0.08), 4SDB (*p* < 0.001, ES = 0.08), and 2.5%SWB (*p* < 0.001, ES = 0.08).

The repeated measures ANOVA for RSImod also showed significant differences existed across the movement thresholds (F = 31.295, *p* < 0.001). Post hoc tests revealed the RSImod for 10%SWAB was greater than the RSImod for 5SDAB (*p* < 0.001, ES = 0.35), 4SDAB (*p* < 0.001, ES = 0.41), 2.5%SWAB (*p* < 0.001, ES = 0.61), 5SDB (*p* < 0.001, ES = 0.30), 4SDB (*p* = 0.002, ES = 0.27), and 2.5%SWB (*p* = 0.001, ES = 0.28). The RSImod for 10%SWB was also greater than the RSImod for 5SDAB (*p* < 0.001, ES = 0.36), 4SDAB (*p* < 0.001, ES = 0.02), 2.5%SWAB (*p* < 0.001, ES = 0.29), 5SDB (*p* < 0.001, ES = 0.30), 4SDB (*p* = 0.001, ES = 0.28), and 2.5%SWB (*p* < 0.001, ES = 0.29). RSImod for 2.5%SWAB was smaller than the RSImod for 5SDB (*p* = 0.017, ES = 0.35), 4SDB (*p* = 0.015, ES = 0.38), and 2.5%SWB (*p* = 0.008, ES = 0.37).

The results from each kinetic and kinematic variable and CV% assessed for each movement initiation method during the SJs are listed in Table 4 and Table 5, respectively. Table 6 lists the ICCs for the SJ data. There were no differences in Fmax across the SJ movement thresholds. The repeated measures ANOVA for netIMP for SJs revealed significant differences existed across the different movement thresholds (F = 6.035, *p* = 0.017). However, our post hoc tests did not reveal any statistical differences.

The repeated measures ANOVA for JHT revealed significant differences existed across the SJ movement thresholds. Post hoc tests showed the JHT for 10%SW was lower than the JHT for 5SD (*p* = 0.005, ES = 0.06) and 4SD (*p* = 0.009, ES = 0.06). JHT for 5SD was higher compared to JHT for 4SD (*p* = 0.005, ES = 0.01).

The repeated measures ANOVA for Pmax revealed significant differences existed across the SJ movement thresholds (F = 5.816, *p* = 0.019). Pmax for 10%SW was lower compared to 5SD (*p* < 0.001, ES = 0.03) and 4SD (*p* = 0.001, ES = 0.03). Pmax for 5SD was greater compared to 4SD (*p* = 0.011, ES < 0.01).

The repeated measures ANOVA for Vmax revealed significant differences existed across the SJ movement thresholds (F = 4.784, *p* = 0.032). Vmax for 10%SW was slower compared to 5SD (*p* = 0.001, ES = 0.07) and 4SD (*p* = 0.005, ES = 0.06). Vmax for 5SD was also faster compared to 4SD (*p* = 0.046, ES 0.01).

## 4. Discussion

The purpose of this investigation was to compare several different movement thresholds when calculating kinetic and kinematic variables during countermovement and squat jumps. Meylan et al. [10] compared different percentages of system weight (2.5%, 5%, and 10%) to assess countermovement jumps. Our findings are similar to their report. We had identified several statistically significant differences among the different countermovement thresholds, however, these differences were fairly small. While Meylan et al. [10] identified the beginning of the movement when the VGRF went below each of their measured thresholds, the current study also identified the first occurrence of when the VGRF went either above or below the threshold. The 2.5%SW and 4SD thresholds retained more of the VGRF than the 5SD and 10%SW thresholds. This is the first study to systematically evaluate methods of movement initiation when the VGRF goes above or below the threshold. In approximately half of all CJs, the earliest detection of movement occurred when the VGRF went above the 2.5%SW threshold. Despite this, the differences across all methods were fairly small. The effect sizes for differences reported in netIMP, brIMP, propulsive impulse, JHT, Pmax, and Vmax for CJs were all trivial (0.02–0.11). The effect sizes for differences in RSImod ranged from trivial to moderate (0.02–0.61). Similarly, the statistically significant differences in JHT, Pmax, and Vmax across the four movement thresholds for SJs were trivial (<0.01–0.07).

Owen et al. [14] evaluated several criteria for determining Pmax during a CJ. One of the criteria was determining when to begin integration of the force-time curve. By plotting the rate of change in standard deviation over time, they identified the beginning of the movement occurring prior to 20 ms before the 5SD threshold. Therefore, they recommended beginning integration 30 ms prior to the 5SD threshold. We did not see any differences in our kinetic and kinematic variables between the 5SD and the 4SD methods. Therefore, using the 4SD method to begin integration may help strength and conditioning professionals simplify their process for measuring CJs on a force plate compared to calculating 5SD and subtracting 30 ms from this time point. While we observed small differences among other methods, the differences observed in netIMP, brIMP, propulsive impulse, JHT, Pmax, and Vmax for CJs may not be practically meaningful. While most effect sizes for RSImod were trivial, there were a few comparisons that resulted in small effect sizes and one moderate effect size. However, caution is warranted when interpreting these comparisons as these measures did not have good absolute reliability as measured by CV%.

All countermovement threshold methods demonstrated good-to-excellent relative reliability (ICC: 0.790–0.990) except for netIMP for 2.5%SWAB (ICC: 0.479). All measures demonstrated acceptable absolute reliability as measured by CV% except for brIMP and RSImod. Our CV% for brIMP were higher than those reported by Lake et al. [13]. They used the 5SD method to compare jump results on a laboratory force plate to a portable force plate. Their CV% for the brIMP on both force plates was 5.0%, whereas the CV% in the present study ranged from 8.5% to 10.4%. Meylan et al. reported similar CV% for the 2.5%SW (8.1%) but lower CV% for 10%SW (5.4%) method compared to the present investigation. While the CV% for most CJ methods for brIMP was <10% in the present study, the 95% CI suggest that several jumpers had a CV% > 10%. Differences in our findings and previous research [9,12] may be attributed to differences in skill level and instructions given to participants. Previous research used athletes whereas the participants of the current study were more heterogenous in their levels of physical activity. In addition, Lake et al. [13]. instructed their participants to move as quickly as possible during the descent of the countermovement to jump as high as possible. Our instructions were to simply jump as high as possible. RSImod also had poor absolute reliability across all methods. Similar to the brIMP findings, this could reflect differences in jumping skill across our subject pool and differences in our instructions compared to previous studies. Therefore, we recommend instructing jumpers to jump for maximum height as quickly as possible in order to reduce the variability in movement time.

As expected, all four methods were able to identify the same peak force since it occurred later in the SJ movement. In addition, there were no differences in netIMP for all four methods. The 5SD and 4SD methods were different from each other and different from 10%SW when assessing JHT, Pmax, and Vmax. While statistically significant, the mean differences between the 5SD and 4SD methods for JHT (0.01 cm), Pmax (4.95 W), and Vmax (<0.01 m/s) were very small. Similarly, the mean differences between 5SD and 10%SW for JHT (0.6 cm), Pmax (37.67 W), and Vmax (0.02 m/s) were also fairly small. The effect sizes for all SJ comparisons were trivial. Therefore, these differences may not be practically meaningful. While most of variables produced good absolute reliability across all methods, the 95% CI for JHT suggests some jumpers had more variability across all five jumps for the 5SD, 4SD, and 2.5%SW methods. This could be due to fatigue and/or differences in skill level. Depsite this, all four methods produced excellent relative reliability for all measures (0.903 to 0.990).

The 2.5%SW method retained more of the force-time curve than any other method while the 10%SW method retained the least amount of the force-time curve. Assessing system weight while in the starting (deep squat) position could have caused an increase in the variability of the VGRF during the weighing phase, which would increase the magnitude of the 5SD and 4SD thresholds. It could also cause the 2.5%SW threshold to identify the jump start too soon, especially in weaker and less skilled participants. This could explain why the CV% for JHT was higher for all four methods when assessing SJs compared to the CV% in CJ. We chose this method for measuring system weight due to the variability in training experience (and presumably strength levels) amongst our subject pool. For JHT, the 10%SW method appeared to produce the best reliability in the present study. Future research may want to evaluate differences in SJ kinetics and kinematics when measuring system weight in a quiet upright stance compared to the deep squat position.

There are some limitations to this study. Our subjects were heterogeneous in terms of their physical activity levels. Therefore, the application of our findings to highly trained athletes may be limited. However, this study’s results may be beneficial to testing and monitoring of novice jumpers. Future research may wish to determine if different populations have different ideal movement thresholds. In assessing SJ performance, system weight was determined with the participant in a self-selected squat position rather than a squat position that had the knee joint at a 90° of knee flexion. While we replicated the self-selected position across all five jumps, assessing system weight during a quiet stance could produce different results. Future research may wish to evaluate these two system weight assessment methods to determine if they have a different impact on the various movement thresholds. While the current study evaluated different methods for identifying the initial movement, another area for future research may wish to explore different methods for identifying take-off.

## 5. Conclusions

While there were statistically significant differences in some CJ metrics across the different movement thresholds, these differences may not be practically meaningful. Interestingly, identifying movement initiation when the VGRF went above or below the threshold compared to when the VGRF went below the threshold resulted in similar reliability for most of the metrics. To improve the reliability for brIMP and RSImod during the CJs, we recommend instructing participants to jump as high and as quickly as possible when using these methods of identifying movement initiation. Differences in the kinetic and kinematic variables analyzed during SJ performance do not appear to be practically meaningful. If measuring system weight in the deep squat position, we recommend using the 10%SW method for identifying the beginning of the movement when analyzing SJs performed on a force plate. Future research may want to evaluate the reliability of these methods when measuring system weight during quiet stance.

## Figures and Tables

**Table 1:** Kinetic and kinematic results for countermovement jump analysis.

Variable	5SDAB	4SDAB	10%SWAB	2.5%SWAB	5SDB	4SDB	10%SWB	2.5%SWB
Fmax (N)	1636.00 (411.53)	1636.00 (411.53)	1636.00 (411.53)	1636.00 (411.53)	1636.00 (411.53)	1636.00 (411.53)	1636.00 (411.53)	1636.00 (411.53)
NetIMP (Ns)	185.93 (58.03)	185.79 (57.70)	186.56 (58.98)	186.57 (58.04)	184.97 (58.335) ^#	184.89 (58.26) ^#	186.38 (59.19)	184.98 (58.19) ^
BrIMP (Ns)	70.30 (24.54)	70.03 (23.98)	68.29 (25.43)	69.67 (24.22)	71.03 (24.80) ^	70.94 (24.79) ^#	68.32 (25.40)	70.86 (24.84) ^#
Propulsive Impulse (Ns)	185.63 (58.03)	185.91 (58.28)	187.64 (59.23)	186.27 (58.00)	184.90 (58.19) ^#	185.00 (58.15) ^#	187.60 (59.27)	185.07 (58.17) ^#
JHT (cm)	29.5 (10.3)	29.6 (10.4)	30.0 (10.6)	29.7 (10.3)	29.1 (10.3) ^#	29.2 (10.3) ^#	30.0 (0.6)	29.2 (10.3) ^#
Pmax (W)	3588.97 (1272.22)	3594.72 (1276.72)	3630.54 (1304.05)	3601.19 (1277.20)	3571.29 (1277.20) ^#	3573.22 (1275.38) ^#	3629.39 (1305.33)	3575.17 (1275.56) ^#
Vmax (m/s)	2.55 (0.39)	2.55 (0.40)	2.57 (0.40)	2.55 (0.39)	2.53 (0.40) ^#	2.53 (0.40) ^#	2.57 (0.40)	2.53 (0.40) ^#
RSImod	0.27 (0.10) ^#	0.26 (0.10) ^#	0.30 (0.12)	0.23 (0.11) ^#	0.27 (0.10) *^#	0.27 (0.10) *^#	0.30 (0.12) *	0.27 (0.10) *^#

Fmax = peak force; NetIMP = net impulse; BrIMP = Braking impulse; JHT = jump height; Pmax = peak power; Vmax = peak velocity. Mean (SD). * significantly different from 2.5%SWAB (*p* < 0.05). ^ significantly different from 10%SWAB (*p* < 0.05). # significantly different from 10%SWB (*p* < 0.05).

**Table 2:** CV% for movement initiation methods for countermovement jumps.

Variable	5SDAB	4SDAB	10%SWAB	2.5%SWAB	5SDB	4SDB	10%SWB	2.5%SWB
Fmax (N)	2.5 [1.741, 3.278]	2.5 [1.741, 3.278]	2.5 [1.741, 3.278]	2.5 [1.741, 3.278]	2.5 [1.741, 3.278]	2.5 [1.741, 3.278]	2.5 [1.741, 3.278]	2.5 [1.741, 3.278]
NetIMP (Ns)	2.8 [2.078, 3.598]	2.8 [2.124, 3.447]	2.8 [2.200, 3.362]	3.1 [1.904, 2.671]	2.6 [2.152, 3.095]	2.7 [2.179, 3.155]	2.7 [2.149, 3.251]	2.6 [2.175, 3.139]
BrIMP (Ns)	9.0 [7.353, 10.590]	9.6 [7.665, 11.592]	10.3 [7.992, 12.712]	9.6 [7.704, 11.582]	8.5 [6.702, 10.337]	9.0 [7.302, 10.593]	10.4 [8.036, 12.736]	8.8 [7.197, 10.403]
Propulsive Impulse (Ns)	2.8 [2.054, 3.470]	3.0 [1.964, 4.094]	2.7 [2.118, 3.215]	3.0 [1.884, 4.050]	2.4 [1.897, 2.826]	2.6 [2.160, 3.106]	2.7 [2.113, 3.210]	2.6 [2.134, 3.095]
JHT (cm)	6.0 [4.125, 7.922]	3.0 [3.806, 9.394]	2.7 [4.364, 7.045]	6.6 [3.776, 9.462]	4.9 [3.998, 5.764]	5.6 [4.379, 6.830]	5.7 [4.336, 7.007]	5.6 [4.332, 6.792]
Pmax (W)	3.2 [2.354, 4.112]	3.5 2.257, 4.781]	3.1 [2.440, 3.817]	3.6 [2.300, 4.852]	2.7 [2.165, 3.273]	3.0 [2.428, 3.639]	3.1 [2.438, 3.810]	3.0 [2.410, 3.638]
Vmax (m/s)	2.3 [2.354, 4.112]	2.5 [1.297, 3.760]	2.2 [2.440, 3.817]	2.6 [1.314, 3.810]	1.8 [2.165, 3.273]	2.1 [1.555, 2.645]	2.1 [1.511, 2.747]	2.1 [1.534, 2.638]
RSImod	8.4 [6.346, 10.568]	11.0 [7.549, 14.375]	8.4 [5.853, 11.014]	15.4 [10.405, 20.452]	8.3 [6.079, 10.492]	9.5 [6.895, 12.038]	8.4 [5.740, 10.965]	10.1 [7.427, 12.859]

Fmax = peak force; NetIMP = net impulse; BrIMP = Braking impulse; JHT = jump height; Pmax = peak power; Vmax = peak velocity. %CV [95% CI].

**Table 3:** ICC [95% CI] for CJ data.

Variable	5SDAB	4SDAB	10%SWAB	2.5%SWAB	5SDB	4SDB	10%SWB	2.5%SWB
Fmax	0.983 [0.968, 0.992]	0.983 [0.968, 0.992]	0.983 [0.968, 0.992]	0.983 [0.968, 0.992]	0.983 [0.969, 0.993]	0.983 [0.968, 0.992]	0.983 [0.968, 0.992]	0.983 [0.968, 0.992]
NetIMP	0.984 [0.970, 0.993]	0.822 [0.698, 0.914]	0.967 [0.938, 0.985]	0.479 [0.278, 0.698]	0.951 [0.909, 0.978]	0.886 [0.798, 0.946]	0.970 [0.943, 0.986]	0.790 [0.651, 0.897]
BrIMP	0.977 [0.957, 0.990]	0.877 [0.785, 0.942]	0.921 [0.857, 0.963]	0.881 [0.790, 0.944]	0.927 [0.868, 0.967]	0.922 [0.858, 0.964]	0.920 [0.855, 0.963]	0.924 [0.862, 0.965]
Propulsive Impulse	0.973 [0.950, 0.988]	0.975 [0.953, 0.989]	0.991 [0.982, 0.996]	0.976 [0.954, 0.989]	0.990 [0.981, 0.995]	0.989 [0.979, 0.955]	0.990 [0.980, 0.995]	0.989 [0.979, 0.995]
JHT	0.932 [0.876, 0.969]	0.878 [0.786, 0.943]	0.969 [0.941, 0.986]	0.879 [0.788, 0.943]	0.958 [0.922, 0.981]	0.954 [0.916, 0.979]	0.961 [0.927, 0.982]	0.954 [0.915, 0.979]
Pmax	0.982 [0.966, 0.992]	0.971 [0.946, 0.987]	0.988 [0.978, 0.995]	0.971 [0.946, 0.987]	0.987 [0.976, 0.994]	0.986 [0.973, 0.994]	0.987 [0.975, 0.994]	0.986 [0.973, 0.994]
Vmax	0.951 [0.910, 0.978]	0.918 [0.852, 0.962]	0.974 [0.952, 0.988]	0.917 [0.850, 0.961]	0.969 [0.942, 0.986]	0.965 [0.935, 0.984]	0.969 [0.942, 0.986]	0.963 [0.932, 0.983]
RSImod	0.923 [0.861, 0.964]	0.873 [0.778, 0.940]	0.931 [0.874, 0.968]	0.804 [0.672, 0.904]	0.901 [0.824, 0.954]	0.853 [0.745, 0.930]	0.922 [0.858, 0.964]	0.879 [0.787, 0.943]

**Table 4:** Kinetic and kinematic results for squat jump analysis.

Variable	5SD	4SD	10%SW	2.5%SW
Fmax (N)	1618.95 (412.02)	1618.95 (412.02)	1618.95 (412.02)	1618.95 (412.02)
NetIMP (Ns)	181.91 (55.98)	181.97 (55.96)	181.29 (56.11)	179.44 (56.08)
JHT (cm)	28.4 (0.96) ^#	28.3 (0.96) ^	27.8 (0.95)	27.7 (0.99)
Pmax (W)	3539.65 (1254.34)^#	3534.70 (1254.59) ^	3501.98 (1249.14)	3493.23 (1261.85)
Vmax (m/s)	2.50 (0.38) ^#	2.50 (0.38) ^	2.48 (0.38)	2.47 (0.40)

Fmax = peak force; NetIMP = net impulse; JHT = jump height; Pmax = peak power; Vmax = peak velocity. Mean (SD). ^ significantly different from 10%SW (*p* < 0.05). # significantly different from 4SD (*p* < 0.05).

**Table 5:** CV% for movement initiation methods for squat jumps.

Variable	5SD	4SD	10%SW	2.5%SW
Fmax (N)	2.2 [1.583, 2.741]	2.2 [1.583, 2.741]	2.2 [1.583, 2.741]	2.2 [1.583, 2.741]
NetIMP (Ns)	3.9 [2.579, 5.830]	3.8 [2.496, 5.123]	3.5 [2.728, 4.205]	3.8 [2.287, 5.218]
JHT (cm)	8.6 [5.369, 11.755]	8.4 [5.225, 11.670]	7.7 [5.880, 9.568]	8.4 [4.952, 11.982]
Pmax (W)	3.9 [2.489, 5.340]	3.9 [2.468, 5.322]	3.5 [2.740, 4.317]	4.1 [2.495, 5.648]
Vmax (m/s)	3.6 [2.255, 4.964]	3.6 [2.257, 4.971]	3.3 [2.488, 4.083]	3.6 [2.145, 5.084]

Fmax = peak force; NetIMP = net impulse; JHT = jump height; Pmax = peak power; Vmax = peak velocity. CV% [95% CI].

**Table 6:** ICC [95% CI] for SJ data.

Variable	5SD	4SD	10%SW	2.5%SW
Fmax	0.990 [0.979, 0.996]	0.990 [0.979, 0.996]	0.990 [0.980, 0.996]	0.989 [0.979, 0.996]
NetIMP	0.984 [0.968, 0.993]	0.985 [0.969, 0.994]	0.984 [0.968, 0.993]	0.981 [0.963, 0.992]
JHT	0.917 [0.843, 0.964]	0.917 [0.844, 0.965]	0.918 [0.845, 0.965]	0.903 [0.819, 0.958]
Pmax	0.986 [0.973, 0.994]	0.987 [0.974, 0.995]	0.986 [0.972, 0.994]	0.983 [0.967, 0.993]
Vmax	0.984 [0.967, 0.993]	0.924 [0.856, 0.968]	0.927 [0.861, 0.969]	0.910 [0.931, 0.961]

## Data Availability

All data are available within the manuscript.
